# Bacterial sulfoquinovose cycling across aquatic ecosystems

**DOI:** 10.1093/femsre/fuag034

**Published:** 2026-07-20

**Authors:** Reagan Lee, Wonjae Kim, Jihye Bae, Woojun Park

**Affiliations:** Laboratory of Molecular Environmental Microbiology, Department of Environmental Science and Ecological Engineering, Korea University, Seoul 02841, Republic of Korea; Laboratory of Molecular Environmental Microbiology, Department of Environmental Science and Ecological Engineering, Korea University, Seoul 02841, Republic of Korea; Laboratory of Molecular Environmental Microbiology, Department of Environmental Science and Ecological Engineering, Korea University, Seoul 02841, Republic of Korea; Laboratory of Molecular Environmental Microbiology, Department of Environmental Science and Ecological Engineering, Korea University, Seoul 02841, Republic of Korea

**Keywords:** freshwater, sulfur cycle, sulfolipid, sulfoquinovose, sulfolysis, cyanobacterial bloom

## Abstract

Sulfoquinovosyldiacylglycerol (SQDG) is a ubiquitous sulfolipid of phototroph membranes, including those of oxygenic phototrophs and some anoxygenic phototrophic bacteria, and its headgroup sulfoquinovose (SQ) represents one of the most abundant organosulfur compounds in the biosphere. Global SQ production is estimated at ∼10 Pg yr^−1^, with marine systems alone contributing ∼1.5 Pg C yr^−1^ in SQ-containing compounds. In marine systems, heterotrophic processing of SQ is increasingly well characterized, whereas the pathways governing SQ transformation in freshwater environments remain poorly resolved. SQ enters both marine and freshwater systems primarily through turnover of phototrophic biomass, with freshwater systems also receiving terrestrial plant-derived sulfolipids. Although phototrophs synthesize SQDG for membrane function, cyanobacteria appear to have limited capacity for SQ catabolism, and SQ turnover is largely mediated by heterotrophic microorganisms. This functional partitioning establishes a metabolic relay in which distinct microbial guilds mediate successive steps of SQ processing, from extracellular sulfolipid hydrolysis to intracellular sulfoglycolysis and terminal sulfur mineralization. Here, we integrate current knowledge of the enzymatic pathways and microbial interactions underpinning SQ transformation within their ecological context. We propose that SQ metabolism constitutes an important, yet underappreciated, axis of aquatic sulfur biogeochemistry, particularly in freshwater systems where cyanobacterial production of dimethylsulfoniopropionate is minimal.

## Teaser

This review synthesizes the enzymatic pathways and microbial interactions underlying sulfoquinovose (SQ) metabolism, arguing that this abundant phototroph-derived sulfolipid headgroup, produced largely by cyanobacteria but catabolized mainly by heterotrophic bacteria through a multistep relay, constitutes an important yet underappreciated axis of aquatic sulfur biogeochemistry that remains poorly resolved, especially in freshwater ecosystems.

## Introduction

**Figure 1:**
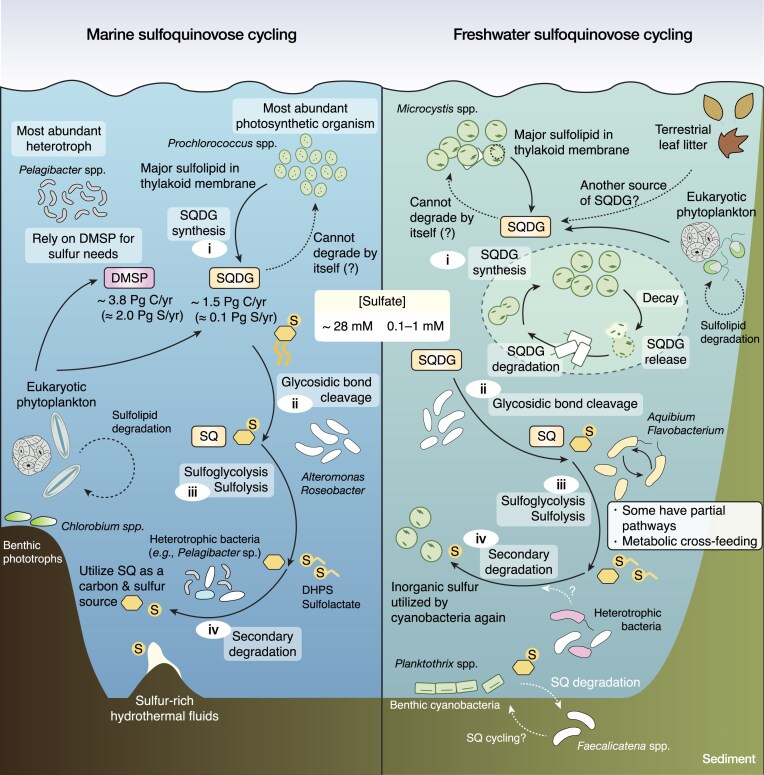
Sulfoquinovose cycling in marine and freshwater aquatic ecosystems. In marine systems (left), where [sulfate] is ∼28 mM, *Prochlorococcus* spp. represent the most abundant photosynthetic organisms and produce SQDG as a major sulfolipid in thylakoid membranes (step i). Total marine production of SQ-containing compounds is estimated at ∼1.5 Pg C yr^−1^ (equivalent to ∼0.1 Pg S yr^−1^), while marine DMSP production is estimated at ∼3.8 Pg C yr^−1^ (equivalent to ∼2.0 Pg S yr^−1^) (Ma et al. 2025). *Pelagibacter* spp., the most abundant marine heterotrophs, rely on DMSP for sulfur needs, while heterotrophic bacteria such as *Alteromonas* and *Roseobacter* carry out glycosidic bond cleavage of SQDG-derived substrates (step ii) and process the released SQ through sulfoglycolysis and sulfolysis (step iii), producing sulfonate intermediates including DHPS and sulfolactate. These sulfonates are utilized as carbon and sulfur sources by secondary degraders (step iv), which regenerate inorganic sulfur. Eukaryotic phytoplankton, including diatoms and green algae such as *Chlamydomonas*, may also contribute to sulfolipid degradation, possessing the enzymatic capacity to potentially recycle their own SQDG. In benthic marine environments, phototrophs such as *Chlorobium* spp. may access sulfur derived from SQ degradation alongside inorganic sulfur from sulfur-rich hydrothermal fluids. In freshwater systems (right), where [sulfate] ranges from 0.1–1 mM, cyanobacterial blooms dominated by genera such as *Microcystis* produce SQDG as a major candidate organic sulfur reservoir in the absence of significant DMSP production. Cyanobacteria may have limited capacity to degrade their own SQDG, and cell decay releases it into the water column, where phycosphere-associated heterotrophic bacteria (potentially including *Sphingomonas, Pseudoduganella, Aquibium*, and *Flavobacterium*) carry out glycosidic bond cleavage (step ii), sulfoglycolysis and sulfolysis (step iii), and secondary degradation (step iv). Some of these organisms harbor individual sulfoglycolytic genes rather than full pathway complements, raising the possibility of metabolic cross-feeding. Secondary degraders further process sulfonate intermediates, regenerating inorganic sulfur that can be reassimilated by cyanobacteria. Allochthonous inputs from terrestrial leaf litter may provide an additional source of SQDG, although its quantitative contribution has not been established. In anoxic sediments, *Faecalicatena* spp. degrade SQ, and benthic cyanobacteria such as *Planktothrix* spp. may access sulfur recycled through SQ degradation, although this coupling has not been directly demonstrated. Dashed arrows and question marks indicate hypothetical or insufficiently characterized steps. The four stages of the SQ metabolic relay are indicated by numbered circles (i through iv). Abbreviations: DMSP, dimethylsulfoniopropionate; DHPS, 2,3-dihydroxypropane-1-sulfonate; SQDG, sulfoquinovosyldiacylglycerol; SQ, sulfoquinovose; Pg, petagrams.

The global sulfur cycle is sustained by a complex interplay between organic and inorganic sulfur pools, the relative contributions of which differ markedly between marine and freshwater ecosystems. Marine and freshwater systems also differ sharply in dissolved sulfate availability, with seawater containing ∼28 mM sulfate and freshwater concentrations typically 100–1000-fold lower (Bueno et al. 2022, Cao et al. 2022, Zhou et al. 2024). In marine environments, organic sulfur cycling is largely driven by dimethylsulfoniopropionate (DMSP), a compatible solute synthesized by algal phytoplankton and utilized by heterotrophic bacteria as a source of carbon and reduced sulfur, with the majority being biologically turned over within days (e.g. 0.6–4.6 days) (Green et al. 2011, Tang and Liu 2023). By contrast, in cyanobacteria-dominated freshwater systems, particularly under eutrophic conditions, cyanobacterial DMSP production appears to be minimal, because cyanobacteria rely on other compatible solutes such as trehalose and sucrose for osmoregulation (Yoch 2002, Hagemann 2011). Sulfonates such as sulfoquinovose (SQ) and 2,3-dihydroxypropane-1-sulfonate (DHPS) represent additional organic sulfur pools in both marine and freshwater systems. The sulfolipid sulfoquinovosyldiacylglycerol (SQDG) is a ubiquitous component of photosynthetic membranes across oxygenic phototrophs, including cyanobacteria (e.g. *Prochlorococcus, Synechocystis*, and *Microcystis*) and vascular plants (Fig. 1, step i) (Villanueva et al. 2014, Goddard-Borger and Williams 2017). Beyond oxygenic phototrophs, SQDG is also present in specific lineages of anoxygenic phototrophic bacteria. Lipid profiling indicates it is present in certain purple bacteria such as *Cereibacter sphaeroides* (previously *Rhodobacter sphaeroides*) and *Rhodovulum sulfidophilum* (Benning and Somerville 1992, Shimada and Takaichi 2024). However, its distribution is not universal, as SQDG has not been detected in green sulfur bacteria or heliobacteria to date (Shimada and Takaichi 2024). In both marine and freshwater conditions, SQDG-derived SQ has also been proposed as an important reservoir of organic sulfur, released from phototrophic membranes and fueling microbial sulfur and carbon metabolism upon degradation (Toda et al. 2023). Although DMSP is regarded as the dominant driver of marine biogenic sulfur cycling, the global abundance of *Prochlorococcus* suggests that cyanobacterial SQDG may also represent a quantitatively important component of marine organic sulfur cycling.

Across oxygenic phototrophs, including cyanobacteria, eukaryotic algae, and vascular plants, SQDG plays a central role in maintaining thylakoid membrane organization and the structural integrity of photosynthetic reaction centers (Hasan and Cramer 2014, Popko et al. 2016, Nakajima et al. 2018). Its sugar headgroup, SQ, is among the most abundant organosulfur compounds in the biosphere, and measurements of SQDG, SQGro, and SQ in marine samples further underscore the substantial contribution of SQ-containing compounds to aquatic carbon and sulfur cycling (Goddard-Borger and Williams 2017, Ma et al. 2025). Current evidence suggests that cyanobacteria have limited capacity to catabolize SQDG-derived SQ (Sato, Kamimura et al. 2017, Ma et al. 2025). Consistent with this, genomic surveys have not identified sulfoquinovosidase homologues, belonging to glycoside hydrolase family 31 (GH31) and glycoside hydrolase family 188 (GH188), within the cyanobacterial phylum. Representative taxa, including *Prochlorococcus, Synechococcus*, and *Synechocystis*, do not exhibit SQDG degradation even under sulfur-limited conditions (Fig. 1, step ii) (Sato, Kamimura et al. 2017, Chen et al. 2025, Ma, Wang, Dong et al. 2025). Notably, it remains unknown whether cyanobacterial lipases can remove the acyl groups from SQDG before sulfoquinovosidase-mediated hydrolysis. In contrast, GH31 and GH188 sulfoquinovosidase orthologs have been identified in diverse eukaryotic algal lineages (Speciale et al. 2016, Kaur et al. 2023, Ma et al. 2025).

In marine systems, the heterotrophic processing of SQ and its downstream sulfonate intermediates is becoming increasingly well characterized (Fig. 1, step iii). Cosmopolitan bacteria, including members of the *Alteromonadaceae* and *Roseobacteraceae*, may contribute substantially to sulfonate-based carbon and sulfur fluxes through SQ catabolism (Ma et al. 2025). The sulfonate intermediates (e.g. 2,3-dihydroxypropane-1-sulfonate, isethionate, sulfolactate) generated by these primary degraders are further processed by downstream consumers. Among these, *Pelagibacter ubique* employs ATP-binding cassette transporters with nanomolar-range substrate affinity, enabling short-chain sulfonate scavenging at sub-micromolar concentrations in oligotrophic waters (Fig. 1, step iv) (Clifton et al. 2024). By contrast, an equivalent ecological framework for SQ cycling in freshwater systems has not yet been established. SQ mineralization in freshwater environments is thought to depend largely on heterotrophic bacteria, with current understanding largely derived from studies of freshwater-recovered SQ-degrading isolates, including *Klebsiella oxytoca, Pseudomonas putida* SQ1, *Bacillus/Priestia* spp., and *Novosphingobium* spp., taxa that are likely of terrestrial origin and therefore may not fully represent bona fide freshwater-adapted microorganisms (Denger et al. 2012, Felux et al. 2015, Liu et al. 2021, Kim et al. 2024a). This division of metabolic roles, in which phototrophs serve as reservoirs of SQDG-derived sulfur and heterotrophic bacteria mediate its turnover, is likely to operate across both marine and freshwater ecosystems, although supporting evidence remains far more developed in marine systems.

Regardless of habitat, SQDG enters aquatic systems primarily through autochthonous production by planktonic and benthic phototrophs (Fig. 1) (Van Mooy et al. 2009, Goddard-Borger and Williams 2017). Phototroph mortality and cellular decay, including bloom decline and virus-associated cell disruption, release cellular material into particulate and dissolved organic matter pools (Pound and Wilhelm 2020, Naknaen et al. 2021, Jung et al. 2022, Kim et al. 2024b). Because SQDG is a membrane-associated sulfolipid, these processes likely release SQDG-derived sulfolipids as part of this material, although the amount released has not been directly quantified. Allochthonous inputs from terrestrial vegetation may provide an additional source, as SQDG-containing chloroplast membranes in higher plants contribute sulfolipids via leaf litter entering streams and littoral zones (Bolik et al. 2022, Cereghetti et al. 2025). Following release, intact SQDG typically requires extracellular processing prior to uptake, as bacteria generally import deacylated intermediates rather than the whole lipid (Fig. 1, step ii) (Liu et al. 2021, Sharma et al. 2021). This functional relationship between phototrophic SQDG producers and heterotrophic SQ degraders can establish a sequential processing relay that may govern organosulfur mobilization in aquatic ecosystems. Addressing current knowledge gaps will require a stepwise resolution of the SQ metabolic relay, beginning with the identification of taxa responsible for sulfolipid degradation and their metabolic capacities, followed by the characterization of pathways underlying sulfoglycolysis and sulfolysis, and culminating in the elucidation of mechanisms driving terminal sulfonate mineralization, particularly in freshwater systems where these processes remain least characterized (Fig. 1, steps iii and iv). An important open question is whether phycosphere-associated bacteria recycle sulfur within cyanobacterial aggregates in ways that may contribute to bloom persistence under sulfate-limited conditions. In this review, we examine cyanobacteria-centered SQ cycling across aquatic ecosystems by comparing marine and freshwater systems. The following sections emphasize cyanobacteria and their associated microbiomes as principal drivers of freshwater SQDG dynamics, while eukaryotic phytoplankton are acknowledged as additional sulfolipid producers. Freshwater ecosystems also encompass diverse benthic and sediment habitats beyond the pelagic water column. These habitats are considered only briefly here, and their microbial pathways and biogeochemical processes warrant dedicated investigation in future studies.

## Biosynthesis, regulation, and function of SQDG in aquatic phototrophs

**Figure 2:**
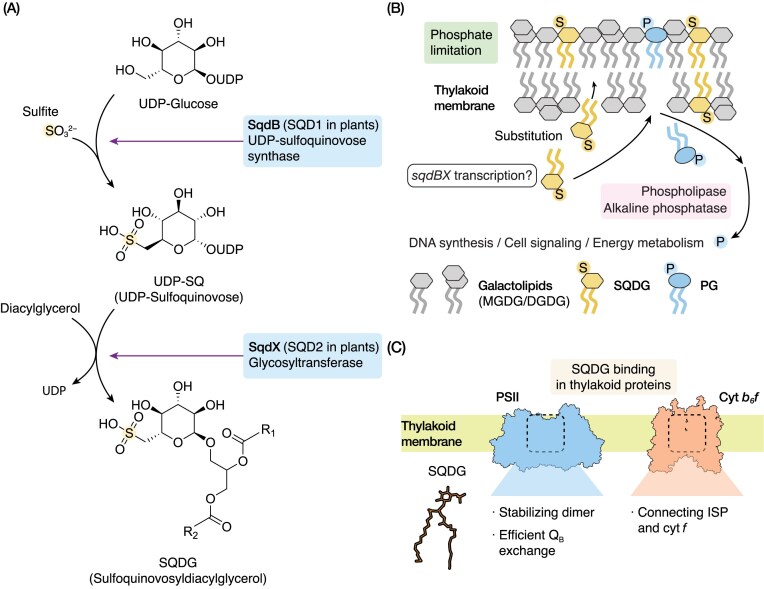
Biosynthesis, structural function, and environmental regulation of SQDG in aquatic phototrophs. (A) The conserved two-step biosynthetic pathway for SQDG production. UDP-sulfoquinovose synthase (SqdB, orthologous to SQD1 in plants) catalyzes the addition of sulfite (SO_3_^2−^) to UDP-glucose, generating UDP-sulfoquinovose (UDP-SQ). The glycosyltransferase SqdX (orthologous to SQD2 in plants) subsequently transfers the sulfoquinovose moiety to a diacylglycerol backbone to produce the mature sulfolipid. Acyl chains are shown as generic R groups representing variable lipoforms. (B) Membrane lipid remodeling under phosphate limitation. Under phosphate-limited conditions, aquatic phototrophs can replace the phospholipid phosphatidylglycerol (PG) with SQDG in their thylakoid membranes, while galactolipids (MGDG/DGDG) remain largely unchanged. Phospholipase and alkaline phosphatase activities liberate phosphorus from degraded PG for reallocation to essential cellular processes including DNA synthesis, cell signaling, and energy metabolism. Whether this substitution triggers *sqdBX* activation is indicated as an open question. (C) Structural roles of SQDG in photosynthetic complexes. SQDG molecules occupy specific binding sites within the photosystem II (PSII) complex, where they stabilize the dimeric structure and facilitate efficient electron transfer at the Q_B_ binding pocket. In the cytochrome *b_6_f* complex (Cyt *b_6_f*), SQDG mediates interactions between the iron-sulfur protein (ISP) and cytochrome *f* on the cytoplasmic side of the thylakoid membrane. Dashed boxes indicate the positions of bound SQDG molecules within each complex. Abbreviations: UDP-SQ, UDP-sulfoquinovose; SQDG, sulfoquinovosyldiacylglycerol; PG, phosphatidylglycerol; MGDG, monogalactosyldiacylglycerol; DGDG, digalactosyldiacylglycerol; PSII, photosystem II; ISP, iron-sulfur protein.

SQDG biosynthesis in model cyanobacteria proceeds via two sequential steps mediated by SqdB and SqdX, with the notable exception of the early-diverging cyanobacterium *Gloeobacter violaceus*, which lacks thylakoid membranes and does not produce SQDG (Fig. 2A) (Selstam and Campbell 1996, Güler et al. 2000, Endo et al. 2016). In the first step, sulfite serves as the sulfur donor, and UDP-sulfoquinovose synthase (SqdB) incorporates it into UDP-glucose to generate UDP-sulfoquinovose (UDP-SQ), thereby establishing the characteristic C–S bond of the sulfolipid headgroup (Villanueva et al. 2014). Subsequently, the glycosyltransferase SqdX transfers the SQ moiety from UDP-SQ to a diacylglycerol backbone, completing SQDG assembly (Güler et al. 2000). The reliance on sulfite as the immediate sulfur donor for UDP-SQ formation has been experimentally demonstrated in plants, green algae, and archaea, but not in cyanobacteria (Sanda et al. 2001, Sato et al. 2003, Zolghadr et al. 2015). The high sequence similarity of cyanobacterial SqdB to its characterized orthologs suggests that sulfite likely serves as the sulfur donor in cyanobacteria as well. The origin of sulfite in cyanobacteria may derive from assimilatory sulfate reduction or the turnover of organosulfonates. In *Arabidopsis thaliana*, the nuclear-encoded and chloroplast-targeted SQD1 and SQD2 are the plant counterparts of cyanobacterial SqdB and SqdX; SQD1 functions as a direct orthologue of SqdB, while SQD2 catalyzes the analogous sulfoquinovosyltransferase step (Benning 1998, Yu et al. 2002, Zimorski et al. 2014). The genomic organization of SQDG biosynthesis genes varies among cyanobacteria. In *Synechococcus elongatus* PCC 7942, *sqdB* and *sqdX* are co-localized and likely co-transcribed, whereas in species such as *Synechocystis* sp. PCC 6803 and *Microcystis aeruginosa* PCC 7806, these genes are separated on the chromosome and may be subject to independent regulatory controls (Sato et al. 2017).

Environmental phosphorus availability is a major determinant of the SQDG-to-PG ratio in thylakoid membranes of marine phytoplankton and picocyanobacteria, operating through a lipid substitution strategy in which phospholipids are replaced by sulfolipids, thereby liberating phosphorus for essential cellular processes such as DNA synthesis, signal transduction, and energy metabolism (Fig. 2B) (Van Mooy et al. 2006, 2009). This response has been observed in both *Prochlorococcus* inhabiting oligotrophic ocean gyres and *M. aeruginosa* populations in Lake Erie, indicating that it represents a broadly conserved adaptation to phosphorus limitation across aquatic ecosystems (Martin et al. 2023, Liu et al. 2025). In plants, phosphate depletion additionally induces the production of glucuronosyldiacylglycerol (GlcADG), a sulfur- and phosphorus-free anionic lipid synthesized by the SQD2 enzyme (Okazaki et al. 2013). Because SQD2 catalyzes both SQDG and GlcADG synthesis, GlcADG production may reflect a broader membrane remodeling response under combined phosphorus and sulfur limitation. While phospholipid-to-sulfolipid substitution under phosphorus limitation is well documented in both marine and freshwater cyanobacteria, the regulatory mechanisms controlling *sqdBX* expression remain poorly defined. Disruption of *sqdBX* affects cell size and growth rate, suggesting that SQDG contributes to cell envelope organization and division in ways that are not explained by its role as a membrane lipid alone (Sato et al. 2017). Notably, despite sulfur limitation constraining SQDG biosynthesis, *sqdB* and *sqdX* are strongly upregulated in *Synechococcus elongatus* PCC 7942, and their deletion results in distinct phenotypes that suggest unexpected functions beyond SQDG production: Δ*sqdB* impairs phycobilisome degradation, whereas Δ*sqdX* causes abnormal chlorophyll loss (Sato et al. 2017).

Beyond its roles in membrane remodeling under nutrient limitation, SQDG is required for the photosynthetic complexes that are not fully compensated by other lipid classes. High-resolution crystallographic analyses of the photosystem II (PSII) complex demonstrate that four SQDG molecules are structurally integrated within each monomer in *Thermosynechococcus elongatus*. Two of these lipids stabilize the dimer at the monomer–monomer interface, while the remaining two are localized near the secondary quinone electron acceptor Q_B_ and between the PsbX and PsbF subunits (Fig. 2C) (Nakajima et al. 2018). Functional studies corroborate this structural role, as loss of SQDG destabilizes the PSII dimer and reduces oxygen evolution, even when other anionic lipids such as phosphatidylglycerol (PG) are supplied as substitutes (Endo et al. 2016). These findings indicate that the sulfoquinovose headgroup provides specific physicochemical properties required for maintaining key structural interactions within PSII. SQDG also occupies specific binding sites in other components of the photosynthetic electron transport chain. In the cytochrome *b_6_f* complex from *Nostoc* PCC 7120, a bound sulfolipid mediates interactions between the Rieske iron–sulfur protein and cytochrome *f*, contributing to structural integrity of the dimeric complex (Fig. 2C) (Hasan and Cramer 2014). The presence of SQDG at specific binding sites in both PSII and the cytochrome *b_6_f* complex suggests that it functions as a structural cofactor rather than a fully interchangeable membrane lipid. This functional specificity likely explains the persistence of SQDG in photosynthetic membranes even when alternative anionic lipids are available. Consequently, SQDG concentrations in aquatic environments are expected to reflect both phototroph abundance and nutrient conditions, particularly phosphorus and sulfur availability. SQDG has established structural roles in thylakoid-embedded photosynthetic complexes, but it remains unclear whether similar structural roles extend to the cyanobacterial cytoplasmic membrane.

## Extracellular SQDG deacylation and SQ liberation

**Figure 3:**
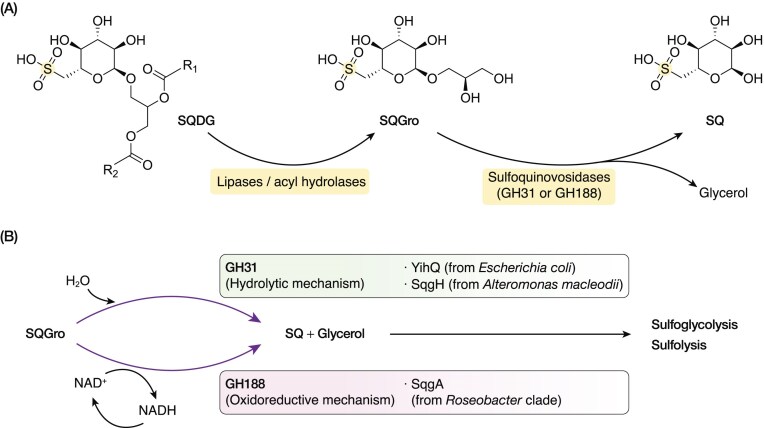
Extracellular processing of SQDG and enzymatic liberation of sulfoquinovose. (A) Stepwise conversion of SQDG to free SQ. Extracellular lipases and acyl hydrolases remove fatty acyl chains (R_1_, R_2_) from SQDG to yield the water-soluble intermediate sulfoquinovosylglycerol (SQGro). Sulfoquinovosidases belonging to either glycoside hydrolase family GH31 or GH188 then cleave the glycosidic bond to release free sulfoquinovose (SQ) and glycerol. (B) Two mechanistically distinct sulfoquinovosidase families catalyze SQGro cleavage. GH31 enzymes employ a hydrolytic mechanism using water as a co-substrate, and include YihQ (characterized in *Escherichia coli*, subfamily GH31_13) and SqgH (characterized in *Alteromonas macleodii*, a distinct GH31 clade). GH188 enzymes (SqgA, characterized in Roseobacter clade bacteria) utilize an NAD^+^-dependent oxidoreductive mechanism in which the cofactor is transiently reduced and regenerated during catalysis. Both families produce the same products (SQ and glycerol), which then enter sulfoglycolytic pathways.

Access to the sulfur and carbon contained in SQDG likely requires extracellular processing of the intact sulfolipid prior to uptake, as bacteria generally import deacylated intermediates rather than the whole lipid (Sharma et al. 2021). This conversion is thought to proceed through at least two sequential steps, in which the lipid backbone is first removed to release the water-soluble intermediate sulfoquinovosylglycerol (SQGro), followed by cleavage of the glycosidic bond to release free SQ (Fig. 3A) (Speciale et al. 2016). Marine bacteria often associated with particulate organic matter have been shown to degrade SQDG derived from phototrophic biomass, indicating that aquatic heterotrophs can access and process exogenous sulfolipid substrates (Ma et al. 2025). Although this deacylation could proceed through conventional lipase activity, the specific enzymes catalyzing this step in aquatic bacteria have not been identified. The second step involves cleavage of the glycosidic bond in SQGro to release free SQ, catalyzed by specialized sulfoquinovosidases belonging to at least two mechanistically distinct families (Fig. 3B). GH31 sulfoquinovosidases, such as SqgH, employ a hydrolytic mechanism and are widely distributed among heterotrophic bacteria, including members of the *Alteromonadaceae* and *Oceanospirillaceae* (Sharma et al. 2021, Ma et al. 2025). In contrast, GH188 enzymes catalyze SQGro cleavage via an NAD^+^-dependent oxidoreductive mechanism. They were first identified in the soil bacteria *Arthrobacter* and *Flavobacterium* and have subsequently been reported in members of the Roseobacter clade, including *Dinoroseobacter shibae* and *Roseobacter denitrificans*, as well as in diverse eukaryotic algal lineages (Kaur et al. 2023). Reflecting these broad phylogenetic assignments, both GH31 and GH188 sulfoquinovosidases are widely distributed across metagenome-assembled genomes (MAGs) of heterotrophic bacteria associated with freshwater and marine phycospheres (Supplementary Table 1). Notably, this two-step model may not be universally required, as the GH31 sulfoquinovosidase YihQ from *Escherichia coli* can hydrolyze intact SQDG directly to release SQ, in addition to cleaving SQGro (Speciale et al. 2016). Whether other sulfoquinovosidases share this dual-substrate capacity, and whether direct SQDG hydrolysis is ecologically relevant in aquatic environments, have not been determined. In marine systems, both GH31 sulfoquinovosidases and GH188 SqgA enzymes are found in diverse eukaryotic algal groups, including diatoms, coccolithophores, dinoflagellates, and red algae, in addition to their established distribution among heterotrophic bacteria (Ma et al. 2025). Some eukaryotic phototrophs also encode GH31 and GH188 homologs, suggesting that they may be capable of recycling their own SQDG under nutrient limitation (Speciale et al. 2016, Kaur et al. 2023). In contrast, none of the cyanobacterial genomes analyzed in this study contained identifiable GH31 or GH188 sulfoquinovosidases (Supplementary Table 1). Variation in the occurrence of sulfoquinovosidase genes across freshwater microbial communities is therefore likely to influence SQDG turnover dynamics, although the link between sulfoquinovosidase gene distribution and actual rates of SQ liberation in freshwater has not been systematically characterized.

## Sulfoglycolysis and sulfonate mineralization in aquatic systems

**Figure 4:**
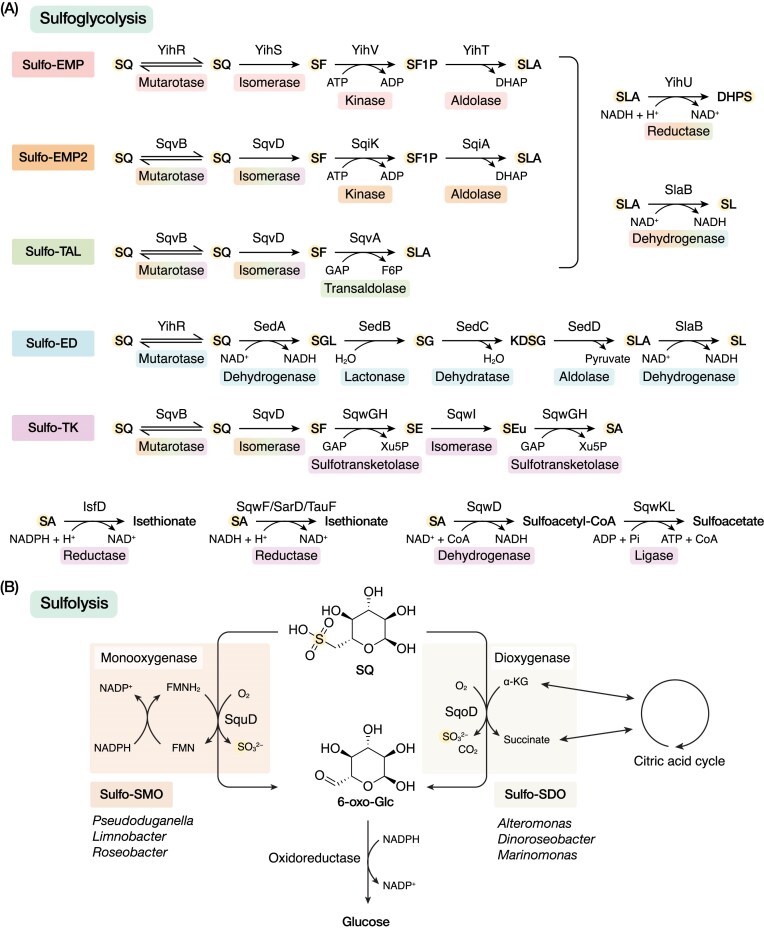
Diversity of sulfoglycolytic pathways for intracellular sulfoquinovose catabolism. (A) Carbon-cleaving sulfoglycolysis routes. Following mutarotase-mediated anomeric conversion, SQ enters divergent pathways that differ in carbon routing, enzyme complement, and sulfonate end products. The sulfo-Embden–Meyerhof–Parnas (sulfo-EMP) pathway proceeds through isomerization to SF, ATP-dependent phosphorylation to SF1P, and aldolase cleavage to yield DHAP and SLA, with SLA reduced to DHPS by YihU. The sulfo-EMP2 variant shares these early steps but uses a distinct kinase (SqiK) and aldolase (SqiA) and oxidizes SLA to SL via the NAD^+^-dependent dehydrogenase SlaB. The sulfo-transaldolase (sulfo-TAL) pathway employs a transaldolase (SqvA) to transfer a three-carbon unit from SF to GAP, yielding SLA and F6P, with SLA oxidized to SL by SlaB. The sulfo-Entner–Doudoroff (sulfo-ED) pathway oxidizes SQ to SGL (SedA), which is hydrolyzed (SedB) and dehydrated (SedC) to KDSG, and then cleaved by the aldolase (SedD) to pyruvate and SLA, with SLA oxidized to SL by SlaB. The sulfo-transketolase (sulfo-TK) pathway channels SF through two successive sulfotransketolase (SqwGH) reactions, each consuming GAP and producing Xu5P, and proceeds via SE and SEu to SA. SA is either reduced to isethionate by SDR-family reductase (IsfD, NADPH-dependent) or an M-ADH-superfamily reductase (SqwF/SarD/TauF, NADH-dependent), or oxidized to sulfoacetyl-CoA (SqwD) and converted to sulfoacetate (SqwKL). SLA represents a central branch point across multiple pathways, with reduction yielding DHPS and oxidation yielding SL. (B) Oxidative sulfolysis pathways that directly cleave the C–S bond of SQ. The sulfo-sulfoquinovose monooxygenase (sulfo-SMO) pathway employs a flavin-dependent monooxygenase (SquD) to convert SQ to 6-oxo-Glc while releasing sulfite (SO_3_^2−^). The sulfo-sulfoquinovose dioxygenase (sulfo-SDO) pathway achieves the analogous conversion using an α-KG-dependent dioxygenase (SqoD), coupling SQ oxidation to α-KG consumption and the production of succinate and CO_2_, which requires regeneration via the citric acid cycle. Both pathways reduce 6-oxo-Glc to glucose via an NADPH-dependent oxidoreductase, recovering the full carbon skeleton for central metabolism. Abbreviations: SF, sulfofructose; SF1P, sulfofructose-1-phosphate; SLA, sulfolactaldehyde; SGL, sulfogluconolactone; SG, sulfogluconate; KDSG, 2-keto-3-deoxy-6-sulfogluconate; DHAP, dihydroxyacetone phosphate; DHPS, 2,3-dihydroxypropane-1-sulfonate; SL, sulfolactate; SA, sulfoacetaldehyde; SE, sulfoerythrose; SEu, sulfoerythrulose; GAP, glyceraldehyde-3-phosphate; F6P, fructose-6-phosphate; Xu5P, xylulose-5-phosphate; 6-oxo-Glc, 6-oxo-glucose; α-KG, α-ketoglutarate.

SQ or SQGro must first be taken up and transported across the cell envelope before intracellular degradation. The best-characterized uptake system is the periplasmic solute-binding protein SmoF and its associated ABC transporter in *Agrobacterium tumefaciens*, with SmoF binding SQGro with sub-micromolar affinity (Sharma et al. 2022, Snow et al. 2022). How aquatic bacteria, particularly those in freshwater environments, acquire SQ-containing substrates remains largely unknown, representing a significant gap in our understanding of environmental SQ cycling. After uptake, SQ enters one of several sulfoglycolytic/sulfolytic routes whose products and energetics differ according to the cleavage strategy employed (Fig. 4). These pathways include sulfo-EMP, sulfo-EMP2, sulfo-ED, sulfo-TAL, sulfo-TK, and the oxidative sulfo-SMO and sulfo-SDO routes (abbreviations defined in the Fig. 4 legend), each producing distinct combinations of short-chain sulfonates and central metabolic intermediates (Fig. 4) (Goddard-Borger and Williams 2017, Abayakoon et al. 2018, Liu et al. 2021, Wei and Zhang 2021, Kaur et al. 2022).

The enzymatic mechanisms of sulfoglycolysis have been elucidated primarily in a limited number of model organisms from gut and soil environments, including *E. coli, Bacillus* spp., and *A. tumefaciens* (Fig. 4) (Denger et al. 2014, Frommeyer et al. 2020, Liu et al. 2021, Sharma et al. 2022). Among these pathways, sulfo-EMP, sulfo-EMP2, and sulfo-TAL all generate sulfolactaldehyde (SLA) as a common intermediate, where reduction yields 2,3-dihydroxypropane-1-sulfonate (DHPS) and oxidation yields sulfolactate (SL), whereas sulfo-ED produces SLA and pyruvate, allowing part of the carbon flux to enter central metabolism directly (Fig. 4A) (Denger et al. 2014, Felux et al. 2015, Liu et al. 2021, Chen et al. 2024, Kaur et al. 2026). The subsequent fate of DHPS and SL varies across organisms, including excretion and further catabolism by the same cell or by secondary degraders. The sulfo-TK pathway channels carbon through a distinct route, ultimately producing isethionate and sulfoacetate as the short-chain sulfonate end products (Krejčík et al. 2010, Zhou et al. 2019, Liu et al. 2021). The oxidative sulfolyase pathways (sulfo-SMO and sulfo-SDO) differ fundamentally in that they are oxygenolytic pathways that cleave the C–S bond directly within a single organism, releasing sulfite and recovering the full carbon skeleton as glucose (Fig. 4B) (Liu et al. 2021, Sharma et al. 2022, Ye et al. 2023, Lee et al. 2026). This direct C–S bond cleavage allows complete SQ mineralization without transfer of organosulfonate intermediates to secondary degraders.

**Figure 5:**
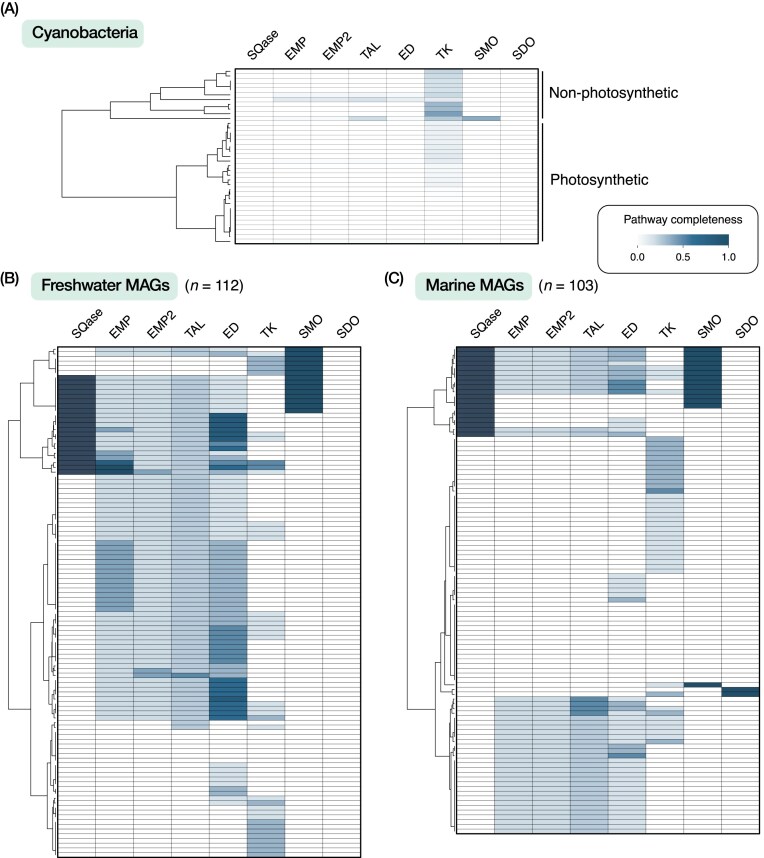
Distribution of sulfoquinovose-degrading capacity across cyanobacterial genomes and MAGs of heterotrophic bacteria associated with phycospheres. Homologs of sulfoquinovosidases and sulfoglycolytic/sulfolytic enzymes were identified by BLASTp (≥ 40% amino-acid identity, ≥ 50% query coverage, *E*-value ≤ 1 × 10^−5^) using verified query sequences for each enzyme. Columns show the sulfoquinovosidase gateway (SQase; GH31 YihQ-type and GH188 SqgA-type) and the completeness of each downstream pathway: sulfo-EMP (EMP), sulfo-EMP2 (EMP2), sulfo-TAL (TAL), sulfo-ED (ED), sulfo-transketolase (TK), and the oxygenolytic sulfo-monooxygenase (SMO) and sulfo-dioxygenase (SDO) routes. Pathway completeness (0–1, shaded scale) is the fraction of required steps detected, with alternative terminal steps counted as satisfied when any one variant is present. (A) Representative cyanobacterial genomes from GTDB (*n* = 3,330), grouped into cyanobacterial orders and arranged by phylogeny, and non-photosynthetic and photosynthetic lineages are indicated. No cyanobacterial genome encoded a credible sulfoquinovosidase of either family above the identity threshold. (B) MAGs of heterotrophic bacteria derived from freshwater cyanobacterial blooms in Lake Taihu, China (*n* = 112). (C) MAGs of heterotrophic bacteria derived from the surface of the Arctic and Atlantic Oceans (*n* = 103). Rows are individual genomes ordered by hierarchical clustering of enzyme profiles. Sulfoquinovosidases were frequently recovered in these heterotrophic bacterial MAGs but not in cyanobacterial genomes, which encoded only a limited set of downstream enzymes. Complete pathways were uncommon among these heterotrophic bacteria, with most genomes encoding only partial gene sets.

In aquatic systems, sulfoglycolysis and short-chain sulfonate mineralization are rarely carried out by a single organism but instead appear to be distributed among different taxa. Sulfoquinovosidases and sulfoglycolytic genes were widely recovered across MAGs of heterotrophic bacteria associated with freshwater and marine phycospheres (Fig. 5, Supplementary Table 1) (Duncan et al. 2022, Cai et al. 2024). Most genomes encode only individual genes rather than the entire pathways necessary for full SQ degradation, suggesting that complete SQ mineralization may depend on metabolic cooperation among co-occurring organisms. However, these incomplete profiles might also stem from fragmented sequence assemblies, the involvement of uncharacterized enzymes, or the possibility that the detected homologs perform unrelated metabolic functions. These genomic patterns are also linked to oxygen availability, as oxic and anoxic environments favor different sulfoglycolytic strategies. Sulfo-EMP predominates in enteric bacteria and is suited to anoxic or microaerobic niches, with strictly anoxic SQ degradation demonstrated in sediment enrichments (Borusak et al. 2024). In contrast, the sulfo-ED is favored under aerobic conditions and was experimentally characterized in freshwater *P. putida* SQ1 (Felux et al. 2015). Oxidative sulfolyase pathways (sulfo-SMO and sulfo-SDO) are typically associated with aerobic taxa, including *Rhizobiales, Oceanospirillaceae*, and select *Gammaproteobacteria* (Liu et al. 2021, Sharma et al. 2022, Ye et al. 2023, Ma et al. 2025). Representative genera include *Roseobacter* for sulfo-SMO and *Alteromonas, Dinoroseobacter*, and *Marinomonas* for sulfo-SDO. The distributed nature of these pathways is consistent with broader public-goods-mediated interactions, including metabolic cross-feeding, in aquatic microbial communities (Park et al. 2025); however, SQ-specific cross-feeding interactions has not yet been tested in aquatic settings (Fig. 1, step iii).

**Figure 6:**
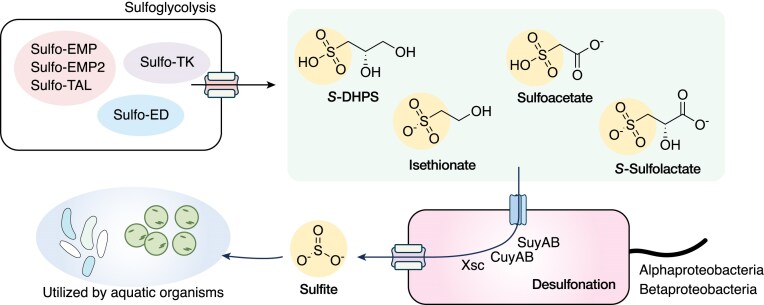
Secondary sulfonate catabolism and terminal desulfonation of sulfonate intermediates produced by sulfoglycolysis pathways. Carbon-cleaving sulfoglycolysis pathways (upper left) export sulfonated end products that serve as substrates for secondary degraders. The sulfo-EMP, sulfo-EMP2, and sulfo-TAL pathways can produce either *S*-DHPS or SL as the primary sulfonate end product, the sulfo-ED pathway produces SL, and the sulfo-TK pathway produces isethionate and sulfoacetate. These sulfonates are taken up by secondary degraders, dominated by select Alphaproteobacteria and Betaproteobacteria (lower right), which carry out terminal C–S bond cleavage through convergent desulfonation routes. The sulfo-lyase SuyAB cleaves sulfolactate to yield pyruvate and sulfite, the thiamine pyrophosphate-dependent acetyltransferase Xsc cleaves sulfoacetaldehyde to yield acetyl phosphate and sulfite, and the pyridoxal phosphate-dependent cysteate sulfo-lyase CuyAB releases pyruvate, ammonium, and sulfite from cysteate. All desulfonation routes converge at sulfite (SO_3_^2−^), which can be oxidized to sulfate and reassimilated by aquatic phototrophs and other organisms (lower left), completing the sulfur cycle. Stereochemical processing is frequently required prior to desulfonation, as sulfoglycolysis typically produces the *S*-enantiomer of DHPS and sulfolactate, whereas terminal desulfonation enzymes such as SuyAB act on the *R*-enantiomer. Only major sulfonate intermediates and desulfonation products are depicted. Abbreviations: DHPS, 2,3-dihydroxypropane-1-sulfonate; SL, sulfolactate; Xsc, sulfoacetaldehyde acetyltransferase; SuyAB, sulfolactate sulfo-lyase; CuyAB, cysteate sulfo-lyase.

Carbon-cleaving sulfoglycolysis does not break the C–S bond but instead yields sulfonated end products such as DHPS, SL, isethionate, and sulfoacetate (Fig. 6). Because these compounds contain chemically stable C–S bonds, their further degradation requires specialized desulfonating enzymes that are phylogenetically restricted and rarely co-inherited with sulfoglycolysis genes (Denger et al. 2014, Wei and Zhang 2021). These short-chain sulfonates are therefore exported and serve as substrates for a specialized community of secondary degraders, dominated by select Alphaproteobacteria and Betaproteobacteria, that carry out C–S bond cleavage through multiple convergent routes, including the 3-sulfolactate sulfo-lyase SuyAB, the sulfoacetaldehyde acetyltransferase Xsc, and the L-cysteate sulfo-lyase CuyAB pathways (Fig. 6) (Denger et al. 2006, 2009, 2012, Mayer et al. 2010, Wei and Zhang 2021). Additionally, glycyl radical enzymes such as HpsHG can cleave DHPS to generate hydroxyacetone and sulfite, providing an alternative anaerobic desulfonation route (Liu et al. 2020). Stereochemical processing is frequently required, as sulfoglycolysis typically produces the *S*-enantiomer of DHPS or SL, whereas terminal desulfonation enzymes act on the *R*-enantiomer (Burchill et al. 2024, Liu et al. 2024). Collectively, the sulfoglycolytic and desulfonation pathways described here can operate across redox gradients spanning oxygenated surface waters to anoxic sediments, positioning SQ-derived short-chain sulfonate metabolism as an active contributor to sulfur cycling in aquatic ecosystems. To our knowledge, outside the oxygenolytic sulfo-SMO and sulfo-SDO routes, complete SQ catabolism by a single organism has not yet been demonstrated, and the reason remains unclear. Maintaining the specialized and energetically demanding enzymes required for C–S bond cleavage may provide little selective advantage when carbon can be recovered without desulfonation. By contrast, organisms occupying sulfur-limited niches may benefit from retaining these enzymes to mineralize smaller sulfonates. These contrasting selective pressures may favor the partitioning of carbon recovery and terminal sulfur mineralization among different organisms. However, this hypothesis remains speculative and requires experimental validation.

## Ecological context of SQ metabolism across aquatic systems

The growth of freshwater phytoplankton is more commonly constrained by the availability of N, P, Si, and trace metals (Huisman et al. 2018). Nevertheless, the development of dense cyanobacterial blooms requires substantial sulfur assimilation. Because freshwater systems generally contain much lower sulfate concentrations than marine environments, the mechanisms that sustain this sulfur demand remain an important ecological question. SQ released from SQDG may contribute to this sulfur cycle by providing a reservoir of organic sulfur and carbon that can be recycled by bloom-associated heterotrophic microorganisms. In marine systems, where sulfate is abundant, SQ-derived sulfonates serve primarily as carbon substrates rather than as critical sulfur sources. Roseobacter clade bacteria have emerged as key participants in this process, with culture-based experiments confirming SQ-dependent growth via the sulfo-SMO pathway and metatranscriptomic data from the Tara Oceans project detecting active *smoC* expression across global ocean provinces (Liu et al. 2023). In some cases, SQ-derived sulfonate exchange is embedded within mutualistic interactions between phototrophs and heterotrophs. A well-characterized example involves diatoms and *Ruegeria pomeroyi* DSS-3, in which diatom-derived SQDG is processed to release DHPS that bacteria utilize as a carbon source, while supplying vitamin B_12_ essential for diatom methionine biosynthesis (Durham et al. 2015).

Freshwater SQ cycling is far less resolved, with current understanding resting primarily on the sulfo-ED pathway in *P. putida* SQ1, isolated from littoral sediments of Lake Constance (Felux et al. 2015, Kaur et al. 2026). This organism retains SQ-derived carbon for growth while excreting SL as a stoichiometric end product, thereby requiring secondary consumers for complete sulfur mineralization. Whether other freshwater bacteria degrade SQ through the same or different sulfoglycolytic routes is unclear. Members of the Bacteroidota, particularly the Flavobacteriaceae, are typically enriched during the early stages of cyanobacterial bloom decomposition and are known to specialize in the degradation of bloom-derived organic matter (Eiler and Bertilsson 2007, Teeling et al. 2012, Kim et al. 2019, Krüger et al. 2019). As noted above, several bloom-associated Bacteroidota also encode sulfoglycolytic gene homologs, raising the possibility that these organisms contribute to the initial processing of SQ-derived substrates released during bloom decline. Under phosphorus-limited conditions, cyanobacteria can increase SQDG production by replacing phospholipids with sulfolipids, thereby reducing cellular phosphorus demand (Van Mooy et al. 2006, 2009). Because cyanobacteria appear to have limited capacity for SQDG-derived SQ catabolism, the turnover of the accumulated SQDG pool may largely depend on heterotrophic microorganisms within the surrounding microbial community (Sato, Kamimura et al. 2017, Ma et al. 2025). Whether heterotrophic bacteria within the phycosphere can locally regenerate sulfate from SQDG released by neighboring cyanobacteria has not been experimentally demonstrated.

In marine systems, SQ degradation occurs primarily in the upper water column, suggesting a tight coupling to phototrophic production and rapid consumption upon release (Ma et al. 2025). By contrast, temporal dynamics of SQ cycling in freshwater are likely tied to bloom progression, with the dominant source of SQDG shifting as blooms develop and decline. Prior to bloom onset, allochthonous inputs of plant-derived SQDG via leaf litter may represent the primary source of organic sulfur entering the system (Yu et al. 2002, Singh et al. 2014, Cereghetti et al. 2025). During active blooms, cyanobacterial biomass is likely to become a dominant source of SQDG, released through cell death (Phillips et al. 2023, Kim et al. 2024c, Kim et al. 2025, Yang et al. 2025). Following bloom senescence, particulate organic matter, including SQDG-containing cell debris, sinks toward sediments; however, the extent to which SQDG accumulates in freshwater sediments, rather than being rapidly mineralized, has not been quantified. In benthic environments, mat-forming cyanobacteria such as *Oscillatoria* and *Phormidium* may access sulfur derived from SQDG degradation alongside inorganic sulfur produced by sulfate-reducing bacteria, thereby linking sulfolipid turnover to benthic primary production (Robichon et al. 2025).

A central unresolved question in both marine and freshwater systems concerns the temporal regulation of sulfoglycolytic activity, as metagenomic surveys document gene presence but cannot distinguish latent capacity from active metabolism (Franzosa et al. 2014). The mismatch between high global SQ production and low environmental concentrations suggests that SQ is rapidly consumed upon release; however, whether capture occurs locally within the phycosphere or after diffusion into bulk water depends on microbial spatial organization and metabolic responsiveness (Nunn et al. 2024). Oxygen availability further determines the ultimate fate of SQ-derived sulfite, as all desulfonation pathways converge at sulfite. This sulfite may be assimilated into cysteine and methionine biosynthesis, or used as a terminal electron acceptor by dissimilatory sulfite-reducing bacteria such as *Desulfovibrio* (Sharma et al. 2022, Ma et al. 2025). Alternatively, it can be oxidized to sulfate by sulfite-oxidizing bacteria such as *Starkeya novella* (Kappler and Dahl 2001). Known sulfite-oxidation systems include the membrane-bound SoeABC complex and periplasmic SorT dehydrogenases. In low-sulfate freshwater systems, sulfate regenerated through sulfonate mineralization may contribute to local replenishment of the dissolved inorganic sulfur pool, whereas in stratified lakes, downward transport of sulfate into anoxic hypolimnia may instead fuel dissimilatory sulfate reduction (Chen et al. 2016, Bueno et al. 2022, Zhou et al. 2024). The balance among these fates varies with stratification, trophic status, and seasonal dynamics, with eutrophic systems likely experiencing greater sulfur sequestration due to high bloom intensity (Baumann et al. 2022, 2024). Although SQDG and SQDG-derived sulfonates represent important components of the aquatic organic sulfur pool, their relative contribution to total sulfur flux remains to be quantified relative to other organosulfur sources such as sulfur-containing amino acids, DMSP, and DHPS.

## Concluding remarks and future perspectives

The central contribution of this review is the recognition that SQ cycling constitutes a consequential, yet underappreciated, axis of sulfur biogeochemistry in aquatic ecosystems. We conceptualize this cycle as a multi-guild metabolic relay defined by four sequential stages (Fig. 1): (i) sulfite assimilation and SQDG biosynthesis via the SqdB–SqdX pathway, (ii) extracellular lipase-mediated deacylation followed by sulfoquinovosidase-catalyzed SQ liberation, (iii) uptake and intracellular sulfoglycolysis by primary degraders, and (iv) terminal desulfonation by secondary degraders to regenerate sulfite. Notably, oxygenolytic pathways such as sulfo-SMO and sulfo-SDO bypass this relay by cleaving the C–S bond directly within a single organism and therefore do not require secondary degraders. While the marine SQ relay is increasingly well resolved, freshwater environments remain largely uncharacterized in terms of the participating taxa, their metabolic interactions, and the quantitative significance of SQ-derived sulfur fluxes. Resolving these uncertainties is particularly important in freshwater systems, where cyanobacterial dominance and limited DMSP production may make SQDG-derived SQ a major reservoir of organic sulfur. SQ-derived carbon may also support heterotrophic microbial metabolism in both marine and freshwater systems, although its quantitative contribution has been better quantified in marine environments. Future research should prioritize integrating time-resolved metatranscriptomics with targeted sulfonate metabolite quantification. Additionally, stable isotope probing combined with targeted sequencing is needed to identify the phylogenetic identity of active SQ consumers, thereby distinguishing primary SQ degraders from secondary short-chain sulfonate consumers. Ultimately, integrating these approaches into dynamic models that incorporate bloom progression, redox gradients, and microbial community structure will be essential for predicting how aquatic SQ metabolism responds to eutrophication, climate-driven changes in stratification, and the increasing global prevalence of cyanobacterial blooms.

## Supplementary Material

fuag034_Supplemental_File
